# Association between modified cardiometabolic index and cardiometabolic multimorbidity in middle-aged and older adults: evidence from two nationwide cohort studies

**DOI:** 10.1038/s41598-026-41398-2

**Published:** 2026-02-23

**Authors:** Shiqin Chen, Tian Lv, Jie Zhou

**Affiliations:** 1 Department of Neurology, Yuhuan Second People’s Hospital, Yuhuan, 317605 China; 2 Department of Neurology, Zhuji Affiliated Hospital of Wenzhou Medical University, Zhuji, 311800 China

**Keywords:** modified cardiometabolic index (MCMI), China Health and Retirement Longitudinal Study (CHARLS), cardiometabolic multimorbidity (CMM), English Longitudinal Study of Ageing (ELSA), insulin resistance, Biomarkers, Cardiology, Diseases, Endocrinology, Medical research, Risk factors

## Abstract

The Modified Cardiometabolic Index (MCMI) is an enhanced version of the Cardiometabolic Index (CMI) and a novel integrative biomarker. Its predictive value for cardiometabolic multimorbidity (CMM), defined as co-occurrence of multiple cardiometabolic conditions, has not been fully explored. We analyzed 7,203 participants from the China Health and Retirement Longitudinal Study (CHARLS, 2011 baseline) and 2,225 from the English Longitudinal Study of Ageing (ELSA, 2012 baseline), with follow-up until 2018 and 2019, respectively. MCMI was calculated as: MCMI = ln [Triglycerides × Fasting Glucose / High-Density Lipoprotein Cholesterol] × Waist Circumference / Height. CMM was defined based on self-reported physician diagnoses of ≥ 2 of the following: hypertension, diabetes, heart disease, or stroke. To assess the association between MCMI and incident CMM, we applied Cox proportional hazards models to estimate hazard ratios (HRs) for time-to-event relationships, restricted cubic spline (RCS) analyses to evaluate potential nonlinear dose–response patterns, and time-dependent receiver operating characteristic (ROC) curves to assess and compare the dynamic predictive performance of MCMI throughout follow-up. Over 7 years, higher MCMI levels were associated with increased CMM risk in both cohorts (CHARLS: HR 1.19, 95% CI 1.16–1.21; ELSA: HR 1.74, 95% CI 1.42–2.12), with risk increasing across quartiles. Participants in the highest quartile had the greatest risk (CHARLS: HR 3.81, 95% CI 3.18–4.56; ELSA: HR 2.77, 95% CI 1.81–4.23). RCS analysis indicated a nonlinear association in CHARLS (*P* < 0.001) and a linear trend in ELSA. Subgroup analyses showed consistent associations across all subgroups in CHARLS, with significantly higher risk observed in older participants, males, those with higher education, smokers, and drinkers (P for interaction < 0.05). In ELSA, associations were consistent except for education, with no significant interactions observed. Time-dependent ROC analysis showed higher area under the curve (AUC) values for MCMI than CMI at 3 and 5 years; DeLong’s test was significant in CHARLS (*P* < 0.05) but not in ELSA. MCMI was positively associated with CMM risk in both cohorts. Its predictive performance was superior to CMI in the CHARLS cohort, whereas no significant difference was observed in ELSA. MCMI may improve clinical risk assessment in the Chinese population, although additional evidence is required to verify its predictive value across different ethnic groups.

**Supplementary Information:**

The online version contains supplementary material available at 10.1038/s41598-026-41398-2.

## Introduction

Cardiometabolic multimorbidity (CMM) refers to the coexistence of two or more cardiometabolic diseases within the same individual, most commonly including hypertension, diabetes, coronary heart disease, and stroke^1,2^. In recent decades, with the continued global rise in obesity and diabetes, the incidence of CMM has increased, and cardiometabolic conditions frequently cluster within middle-aged and older adults^3–5^. Individuals with CMM face significantly higher risks of all-cause and cardiovascular mortality, shorter life expectancy, and greater disability and reduced quality of life, such as functional limitation from heart failure, stroke sequelae, and diabetes complications, while also generating higher healthcare utilization including emergency visits, hospitalizations, and long-term care^2,6–9^. Given the substantial health and societal burden of CMM, identifying modifiable risk factors is particularly important. Evidence suggests that adiposity and metabolic markers, such as body mass index, waist circumference, and the triglyceride–glucose index, are closely associated with the development of CMM^10,11^.

The cardiometabolic index (CMI), a composite indicator reflecting visceral adiposity and lipid metabolism abnormalities, is calculated from the waist-to-height ratio (WC/height), triglycerides (TG), and high-density lipoprotein cholesterol (HDL-C). CMI was first proposed by Wakabayashi and Daimon in 2015 as a simplified surrogate marker to identify individuals at increased risk of type 2 diabetes by capturing central obesity–related insulin resistance and atherogenic dyslipidemia^12^. Since its introduction, CMI has received growing attention in cardiometabolic research because it integrates anthropometric and lipid parameters and is simple to obtain in clinical settings. Subsequent studies have demonstrated that CMI is independently associated with multiple cardiometabolic disorders, including cardiovascular disease and stroke^13,14^, and observational evidence has further linked higher CMI levels to an elevated CMM risk^15^. However, the predictive ability of CMI remains limited by its reliance solely on lipid-related markers and the absence of direct glycemic components, despite insulin resistance being a central mechanism underlying the development of metabolic syndrome and cardiometabolic diseases.

To address these limitations, Guo et al. recently proposed the modified cardiometabolic index (MCMI), which incorporates fasting blood glucose (FBG) into the original CMI formula to enhance its ability to capture insulin resistance and glucose–lipid metabolic interactions^16^. The inclusion of FBG is particularly important because impaired glucose metabolism and compensatory hyperinsulinemia represent early pathophysiological changes that precede overt cardiometabolic diseases and contribute to the clustering of metabolic abnormalities. Emerging evidence suggests that MCMI outperforms CMI in predicting metabolic dysfunction–related disorders, such as non-alcoholic fatty liver disease (NAFLD) and liver fibrosis^16^. However, to date, no study has evaluated the association between MCMI and CMM, nor compared the predictive performance of MCMI versus CMI in the development of cardiometabolic multimorbidity. In addition, limited evidence exists regarding whether the predictive value of MCMI is consistent across populations of different ethnic backgrounds. Although both CMI and MCMI were originally developed and validated primarily in Asian populations, where visceral adiposity and insulin resistance patterns differ from Western populations, it remains unclear whether MCMI retains its discriminatory ability outside Asian settings.

Therefore, leveraging two nationally representative longitudinal cohorts—the China Health and Retirement Longitudinal Study (CHARLS, 2011–2018) and the English Longitudinal Study of Ageing (ELSA, 2012–2019)—the present study aimed to examine the association between MCMI and incident CMM and to compare the predictive utility of MCMI with that of CMI. The inclusion of two ethnically and geographically distinct cohorts provides an opportunity to evaluate the generalizability and robustness of MCMI across diverse populations and to determine whether a metabolic index originally developed in Asian populations can be reliably applied to Western settings.

## Methods

### Study population

CHARLS and ELSA are nationally representative longitudinal surveys targeting middle-aged and older adults, designed to collect comprehensive information on demographics, socioeconomic status, health conditions, and medical service utilization^17,18^. CHARLS employed a multistage, stratified, probability-proportional-to-size sampling strategy to select participants from 150 county-level units across 28 provinces in mainland China. Individuals aged ≥ 45 years were recruited, and follow-up interviews have been conducted every 2–3 years since 2011^17^. ELSA, conducted in the United Kingdom, used a multistage stratified sampling design based on the Health Survey for England (HSE) sampling frame. Individuals aged ≥ 50 years living in private households across England were recruited, with biennial follow-up waves initiated in 2002^18^. Both surveys are therefore considered representative of their respective national populations. Protocols were approved by the Ethics Committee of Peking University (CHARLS) and the London Multicentre Research Ethics Committee (ELSA), and all participants provided written informed consent.

**Fig. 1:**
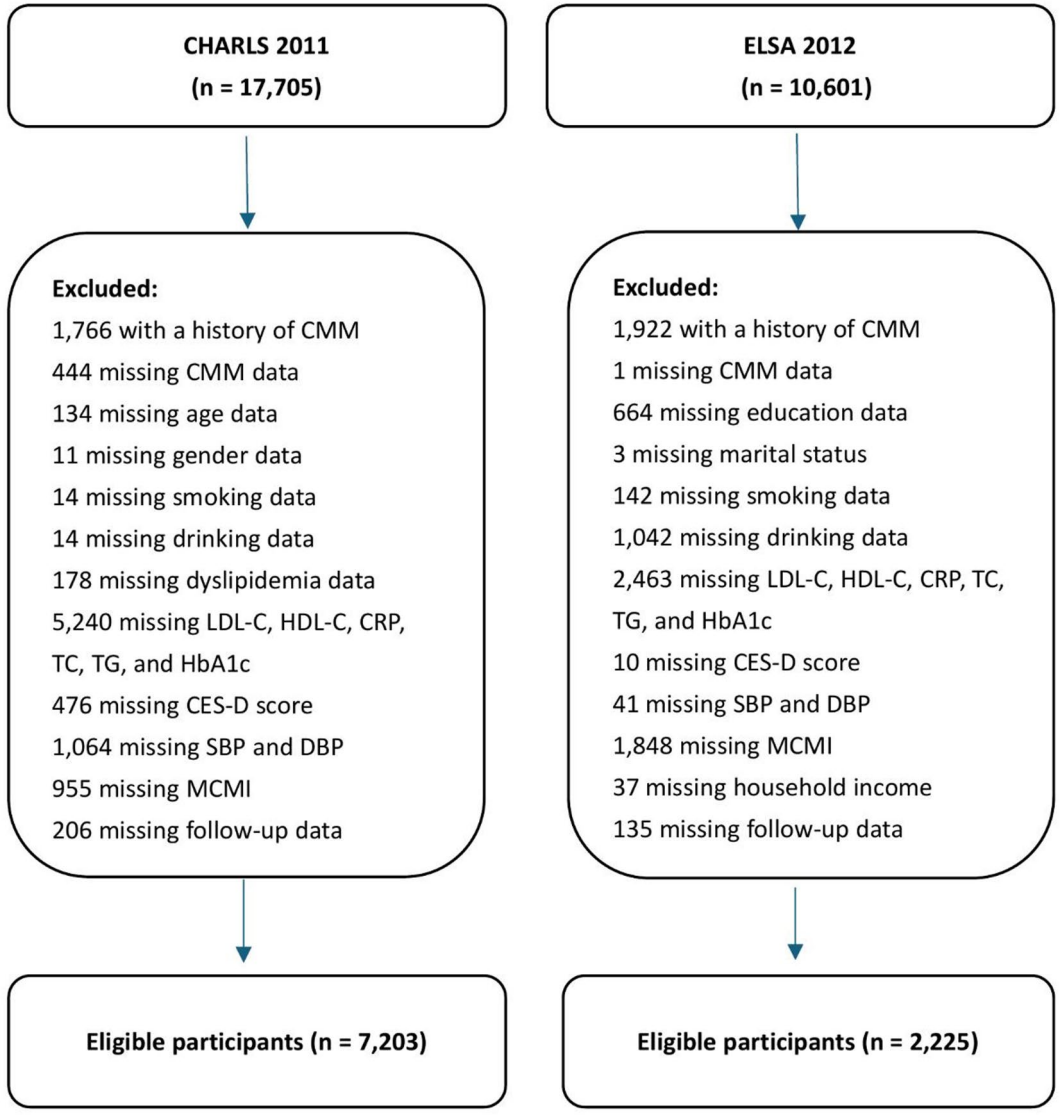
Flowchart of the study population. Abbreviations: CHARLS, China Health and Retirement Longitudinal Study; ELSA, English Longitudinal Study of Ageing; CMM, cardiometabolic multimorbidity; HbA1c, glycated hemoglobin; CRP, C-reactive protein; LDL-C, low-density lipoprotein cholesterol; HDL-C, high-density lipoprotein cholesterol; TC, total cholesterol; TG, triglycerides; SBP, systolic blood pressure; DBP, diastolic blood pressure; CES-D, Center for Epidemiologic Studies Depression Scale; MCMI, modified cardiometabolic index.

To enhance comparability in temporal alignment and data structure rather than ethnic composition, we selected harmonized baseline years: wave 1 of CHARLS (2011) and wave 6 of ELSA (2012), both of which included standardized physical examinations and laboratory testing. Participants were followed until 2018 in CHARLS and 2019 in ELSA. Individuals were eligible if they had complete baseline demographic, socioeconomic, health, anthropometric, and laboratory data. Participants with CMM at baseline or missing follow-up information were excluded. A total of 7,203 participants from CHARLS and 2,225 from ELSA were included in the analytic sample (Fig. 1).

Although CHARLS and ELSA represent different ethnic populations, both are internationally recognized harmonized ageing cohorts widely used for cross-country comparative studies^19,20^. Using two ethnically distinct, nationally representative cohorts allows assessment of the robustness and generalizability of MCMI across diverse populations, which aligns with the objective of validating a metabolic index beyond a single ethnic context.

### Calculation of MCMI and CMI

CMI and MCMI were calculated as previously described^16^. The formulas are expressed as follows:

$$\:CMI=\frac{TG\left(mmol/L\right)}{HDL-C\:\left(mmol/L\right)}\:\times\:\:\frac{WC\:\left(cm\right)}{Height\:\left(cm\right)}$$

$$\:MCMI=\mathrm{l}\mathrm{n}\left(\frac{TG\:\left(mg/dL\right)\times\:FBG\:(mg/dL)}{HDL-C\:\left(mg/dL\right)}\right)\:\times\:\:\frac{WC\:\left(cm\right)}{Height\:\left(cm\right)}$$

In these formulas, TG represents triglycerides; HDL-C, high-density lipoprotein cholesterol; WC, waist circumference; Height, body height; and FBG, fasting blood glucose. Note that CMI uses TG and HDL-C in mmol/L, whereas MCMI follows its original definition using TG and FBG in mg/dL.

### Follow-up of CMM events

The primary outcome was the incidence of CMM. CMM was ascertained through self-reported physician diagnoses of hypertension, diabetes, heart disease, or stroke using a standardized questionnaire, a method that has been widely applied in previous studies^1,21^. The onset of CMM was defined as the date of the first reported new diagnosis during the follow-up period.

### Covariates

Based on previous studies^1,22^, covariates were collected at baseline, including demographic, socioeconomic, health-related, and laboratory data. Demographic and socioeconomic variables were assessed, including age, sex, marital status, educational attainment, and household income (couple level, as reported in the survey). Health-related variables included self-reported smoking and drinking status, dyslipidemia (self-reported physician diagnosis), and depressive symptoms. Depressive symptoms were assessed using cohort-specific, validated short-form Center for Epidemiologic Studies Depression scales (CES-D): CES-D10 in CHARLS (score ≥ 10) and CES-D8 in ELSA (score ≥ 4)^23,24^. Although the scoring systems differ, both instruments are widely used in international ageing cohorts and are recommended for cross-country epidemiological analyses^25,26^. Laboratory assessments comprised glycated hemoglobin (HbA1c), low-density lipoprotein cholesterol (LDL-C), and C-reactive protein (CRP).

### Statistical analysis

Continuous variables were compared using independent samples t-tests or Mann–Whitney U tests and presented as mean ± standard deviation (SD) or median with interquartile range (IQR), as appropriate. Categorical variables were compared using the chi-square test and reported as frequencies with percentages.

To investigate the longitudinal association between MCMI and the incidence of CMM, we constructed three Cox proportional hazards regression models. Cox models are appropriate for time-to-event analyses, allowing estimation of hazard ratios (HRs) while accounting for censoring. The models were specified as follows:

Model 1: unadjusted;

Model 2: adjusted for age, sex, marital status, educational attainment, smoking, and drinking;

Model 3: additionally adjusted for household income, LDL-C, CES-D score, and CRP.

Missing data were handled by excluding participants with incomplete covariate or outcome information.

Further analyses categorized MCMI into quartiles, and cumulative incidence was assessed using Kaplan–Meier curves. Restricted cubic spline (RCS) models were applied to explore potential nonlinear dose–response relationships between MCMI and CMM risk.

Subgroup analyses were performed to assess robustness and potential effect modification by age, sex, education, marital status, smoking, and drinking. To assess the predictive performance of MCMI, we applied time-dependent receiver operating characteristic (ROC) curves, which allow evaluation of discrimination at different follow-up times in the presence of censoring. The area under the curve (AUC) was calculated, and predictive ability between MCMI and CMI was compared using the DeLong test.

Sensitivity analyses included calculation of the E-value to quantify the potential impact of unmeasured confounding^27^.

All statistical analyses were conducted in R software (version 4.4.2), and a two-sided P value < 0.05 was considered statistically significant.

## Results

### Baseline characteristics

During the 7-year follow-up, 7,203 participants from CHARLS and 2,225 from ELSA were included. The incidence of CMM was 17.7% and 10.2%, respectively. Participants were stratified by CMM status. Compared with the non-CMM group, those who developed CMM were older (CHARLS: 60.3 vs. 57.8 years; ELSA: 66.8 vs. 64.6 years), had higher HbA1c (CHARLS: 5.5 vs. 5.2; ELSA: 5.9 vs. 5.7), CRP (CHARLS: 1.3 vs. 0.9; ELSA: 1.8 vs. 1.3), and MCMI levels (CHARLS: 3.2 vs. 2.8; ELSA: 3.1 vs. 2.8). In addition, the prevalence of dyslipidemia (CHARLS: 14.2% vs. 6.0%; ELSA: 46.0% vs. 33.2%) and depressive symptoms (CES-D ≥ cut-off; CHARLS: 43.0% vs. 34.8%; ELSA: 15.9% vs. 8.2%) was higher in the CMM group. Detailed baseline characteristics are shown in Table 1.

**Table 1:** Baseline characteristics of participants by CMM status.

Characteristic	Levels	Non-CMM (CHARLS)	CMM (CHARLS)	*P* value	Non-CMM (ELSA)	CMM (ELSA)	*P* value
N		5927	1276		1999	226	
Age (mean (SD))		57.8 (9.6)	60.3 (8.9)	< 0.001	64.6 (7.3)	66.8 (7.3)	< 0.001
Gender (%)	Female	3153 (53.2)	741 (58.1)	0.002	1163 (58.2)	121 (53.5)	0.205
	Male	2774 (46.8)	535 (41.9)		836 (41.8)	105 (46.5)	
Education (%)	Below high school	5341 (90.1)	1160 (90.9)	0.414	517 (25.9)	61 (27.0)	0.774
	High school or above	586 ( 9.9)	116 ( 9.1)		1482 (74.1)	165 (73.0)	
Marital status	Married	5296 (89.4)	1102 (86.4)	0.002	1544 (77.2)	171 (75.7)	0.652
	Single	631 (10.6)	174 (13.6)		455 (22.8)	55 (24.3)	
Smoking (%)	No	3600 (60.7)	825 (64.7)	0.010	808 (40.4)	90 (39.8)	0.919
	Yes	2327 (39.3)	451 (35.3)		1191 (59.6)	136 (60.2)	
Drinking (%)	No	3586 (60.5)	792 (62.1)	0.314	156 ( 7.8)	31 (13.7)	0.004
	Yes	2341 (39.5)	484 (37.9)		1843 (92.2)	195 (86.3)	
HbA1c (mean (SD))		5.2 (0.6)	5.5 (1.0)	< 0.001	5.7 (0.4)	5.9 (0.6)	< 0.001
LDL-C (mean (SD), mmol/L)		3.0 (0.9)	3.1 (1.0)	< 0.001	3.4 (1.0)	3.3 (1.1)	0.036
HDL-C (mean (SD), mmol/L)		1.4 (0.4)	1.3 (0.4)	< 0.001	1.7 (0.5)	1.6 (0.5)	0.007
TC (mean (SD), mmol/L)		5.0 (1.0)	5.1 (1.0)	< 0.001	5.8 (1.1)	5.6 (1.2)	0.025
CRP (median [IQR], mg/L)		0.9 [0.5, 1.9]	1.3 [0.7, 2.7]	< 0.001	1.3 [0.7, 2.6]	1.8 [1.0, 3.5]	< 0.001
TG (mean (SD), mmol/L)		1.4 (1.0)	1.6 (1.1)	< 0.001	1.3 (0.6)	1.4 (0.7)	0.003
CMI (median [IQR])		0.4 [0.3, 0.8]	0.6 [0.4, 1.0]	< 0.001	0.4 [0.2, 0.6]	0.5 [0.3, 0.7]	< 0.001
MCMI (mean (SD))		2.8 (0.7)	3.2 (1.1)	< 0.001	2.8 (0.6)	3.1 (0.6)	< 0.001
Dyslipidemia (%)	No	5572 (94.0)	1095 (85.8)	< 0.001	1336 (66.8)	122 (54.0)	< 0.001
	Yes	355 ( 6.0)	181 (14.2)		663 (33.2)	104 (46.0)	
CES-D score (%)	Depressive symptoms	2065 (34.8)	549 (43.0)	< 0.001	164 ( 8.2)	36 (15.9)	< 0.001
	No depressive symptoms	3862 (65.2)	727 (57.0)		1835 (91.8)	190 (84.1)	
Household income (median [IQR], GBP)		1680.0 [0.0, 18037.5]	1320.0 [0.0, 13464.0]	0.007	25946.9 [16751.8, 39692.3]	25622.4 [16393.7, 38328.8]	0.664
SBP (mean (SD), mmHg)		125.7 (20.1)	137.0 (20.9)	< 0.001	130.6 (16.7)	137.3 (17.1)	< 0.001
DBP (mean (SD), mmHg)		73.8 (11.7)	78.8 (12.3)	< 0.001	75.1 (9.7)	76.6 (10.3)	0.024
Quartiles of MCMI (%)	Q1	1638 (27.6)	163 (12.8)	< 0.001	524 (26.2)	32 (14.2)	< 0.001
	Q2	1547 (26.1)	253 (19.8)		508 (25.4)	48 (21.2)	
	Q3	1479 (25.0)	322 (25.2)		497 (24.9)	59 (26.1)	
	Q4	1263 (21.3)	538 (42.2)		470 (23.5)	87 (38.5)	

Abbreviations: CHARLS, China Health and Retirement Longitudinal Study; ELSA, English Longitudinal Study of Ageing; CMM, cardiometabolic multimorbidity; HbA1c, glycated hemoglobin; LDL-C, low-density lipoprotein cholesterol; HDL-C, high-density lipoprotein cholesterol; TC, total cholesterol; TG, triglycerides; CRP, C-reactive protein; SBP, systolic blood pressure; DBP, diastolic blood pressure; CES-D, Center for Epidemiologic Studies Depression Scale; MCMI, modified cardiometabolic index; CMI, cardiometabolic index.

### Association between MCMI and CMM risk

**Fig. 2:**
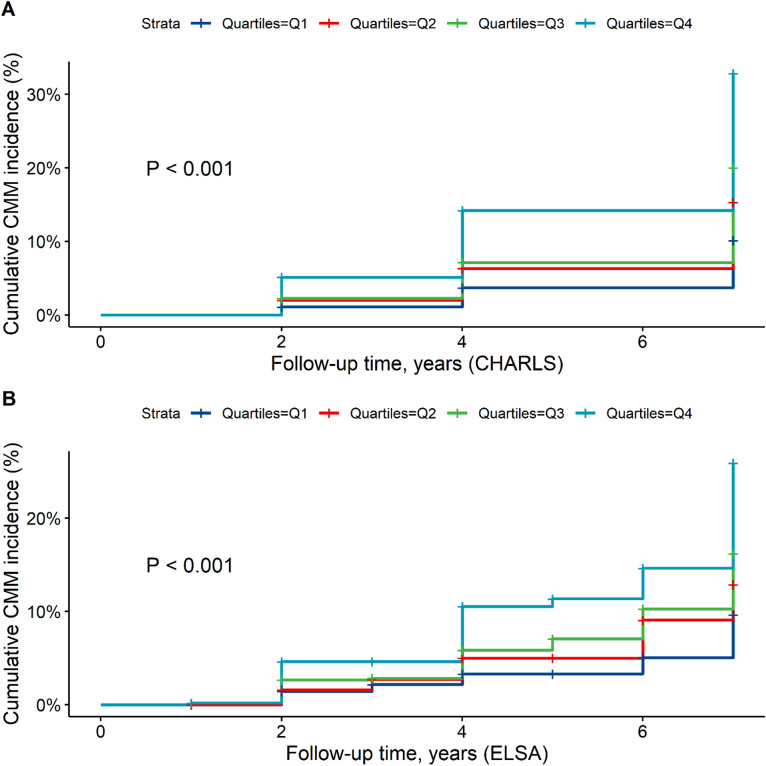
Kaplan–Meier analysis of CMM incidence across MCMI quartiles. Abbreviations: CHARLS, China Health and Retirement Longitudinal Study; ELSA, English Longitudinal Study of Ageing; CMM, cardiometabolic multimorbidity.

In the fully adjusted model (Model 3), higher MCMI levels were significantly associated with an increased risk of CMM in both cohorts (CHARLS: HR = 1.19, 95% CI: 1.16–1.21; ELSA: HR = 1.74, 95% CI: 1.42–2.12) (Table 2). When categorized by quartiles of MCMI, a stepwise increase in CMM risk was observed compared with the lowest quartile (Q1): Q2 (CHARLS: HR = 1.58, 95% CI: 1.29–1.92; ELSA: HR = 1.50, 95% CI: 0.95–2.38), Q3 (CHARLS: HR = 2.06, 95% CI: 1.71–2.50; ELSA: HR = 1.83, 95% CI: 1.17–2.86), and Q4 (CHARLS: HR = 3.81, 95% CI: 3.18–4.56; ELSA: HR = 2.77, 95% CI: 1.81–4.23). A significant increasing trend in CMM risk was observed across quartiles of MCMI (P for trend < 0.001). Consistently, Kaplan–Meier curves (Fig. 2) demonstrated higher cumulative incidence of CMM among participants in the highest quartile, with significant group differences (log-rank *P* < 0.001).

**Table 2:** Sensitivity analyses of the association between MCMI and CMM risk Model 1: Unadjusted. Model 2: Adjusted for age, sex, marital status, educational attainment, smoking, drinking. Model 3: Additionally adjusted for household income, LDL-C, CES-D score, and CRP.

Variable	Model1 HR(95% CI)	*P* value	Model2 HR(95% CI)	*P* value	Model3 HR(95% CI)	*P* value
**CHARLS**						
MCMI (pre 1-unit)	1.17 (1.14, 1.19)	< 0.001	1.18 (1.15, 1.20)	< 0.001	1.19 (1.16, 1.21)	< 0.001
Quartiles of MCMI						
Q1 (MCMI < 2.42)	Ref		Ref		Ref	
Q2 (2.42 ≤ MCMI < 2.83)	1.58 (1.29, 1.92)	< 0.001	1.58 (1.30, 1.93)	< 0.001	1.58 (1.29, 1.92)	< 0.001
Q3 (2.83 ≤ MCMI < 3.31)	2.05 (1.70, 2.48)	< 0.001	2.08 (1.72, 2.52)	< 0.001	2.06 (1.71, 2.50)	< 0.001
Q4 (MCMI ≥ 3.31)	3.74 (3.14, 4.46)	< 0.001	3.73 (3.12, 4.46)	< 0.001	3.81 (3.18, 4.56)	< 0.001
P for trend		< 0.001		< 0.001		< 0.001
**ELSA**						
MCMI (pre 1-unit)	1.81 (1.49, 2.20)	< 0.001	1.78 (1.45, 2.18)	< 0.001	1.74 (1.42, 2.12)	< 0.001
Quartiles of MCMI						
Q1 (MCMI < 2.41)	Ref		Ref		Ref	
Q2 (2.41 ≤ MCMI < 2.84)	1.61 (1.02, 2.52)	0.039	1.48 (0.93, 2.33)	0.095	1.50 (0.95, 2.38)	0.084
Q3 (2.84 ≤ MCMI < 3.25)	1.94 (1.26, 3.00)	0.003	1.79 (1.15, 2.79)	0.01	1.83 (1.17, 2.86)	0.008
Q4 (MCMI ≥ 3.25)	3.01 (2.00, 4.53)	< 0.001	2.76 (1.81, 4.21)	< 0.001	2.77 (1.81, 4.23)	< 0.001
P for trend		< 0.001		< 0.001		< 0.001

Abbreviations: CHARLS, China Health and Retirement Longitudinal Study; ELSA, English Longitudinal Study of Ageing; CMM, cardiometabolic multimorbidity; LDL-C, low-density lipoprotein cholesterol; CES-D, Center for Epidemiologic Studies Depression Scale; CRP, C-reactive protein; MCMI, modified cardiometabolic index; HR, hazard ratio; CI, confidence interval.

### Dose–response relationship between MCMI and CMM

**Fig. 3:**
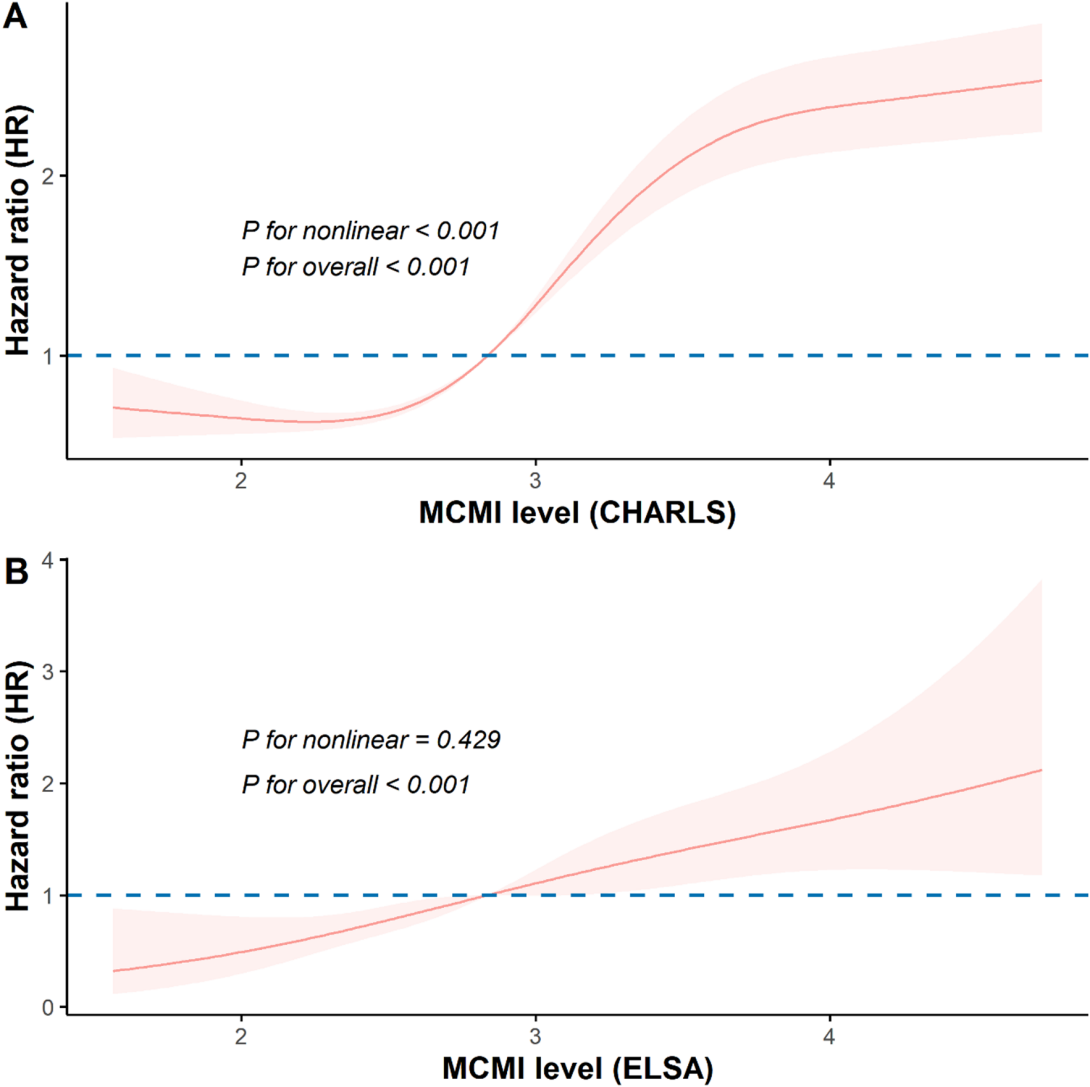
Dose–response relationship between MCMI and CMM risk. Restricted cubic spline showing adjusted hazard ratios for CMM across MCMI levels. The model was adjusted for age, sex, marital status, educational attainment, smoking, drinking, household income, LDL-C, CES-D score, and CRP. Abbreviations: CHARLS, China Health and Retirement Longitudinal Study; ELSA, English Longitudinal Study of Ageing; CMM, cardiometabolic multimorbidity; MCMI, modified cardiometabolic index; LDL-C, low-density lipoprotein cholesterol; CES-D, Center for Epidemiologic Studies Depression Scale; CRP, C-reactive protein.

RCS analysis revealed a significant overall association between MCMI and CMM risk in the CHARLS cohort (P for overall < 0.001), with evidence of a nonlinear relationship (P for nonlinear < 0.001). In contrast, in the ELSA cohort, the association appeared linear (P for overall < 0.001; P for nonlinear = 0.429, not statistically significant). These results indicate a potential cohort-specific difference in the dose–response relationship of MCMI with CMM risk (Fig. 3).

### Subgroup analyses

**Fig. 4:**
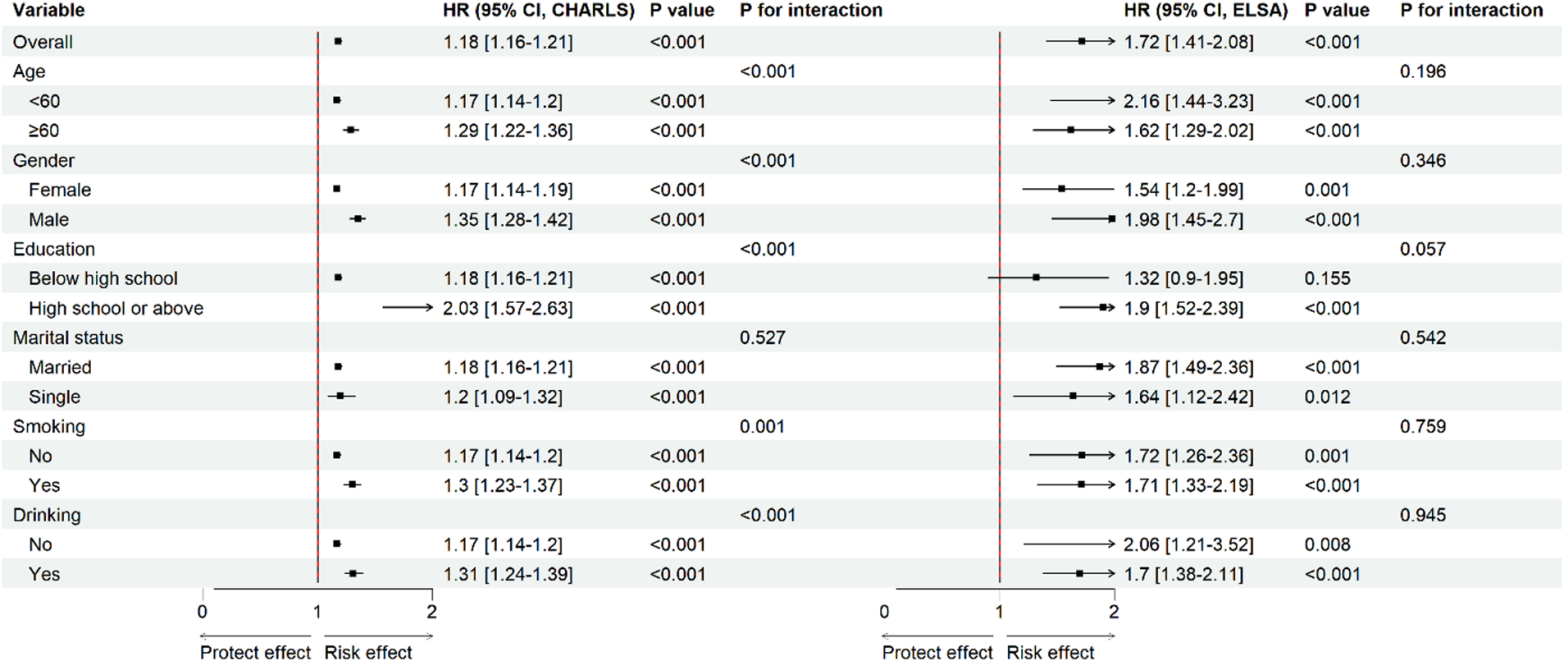
Subgroup analyses of CMM risk by MCMI. Forest plot showing hazard ratios and 95% confidence intervals for CMM risk across subgroups defined by MCMI. The model was adjusted for age, sex, marital status, educational attainment, smoking, drinking, household income, LDL-C, CES-D score, and CRP. Abbreviations: CHARLS, China Health and Retirement Longitudinal Study; ELSA, English Longitudinal Study of Ageing; CMM, cardiometabolic multimorbidity; MCMI, modified cardiometabolic index; LDL-C, low-density lipoprotein cholesterol; CES-D, Center for Epidemiologic Studies Depression Scale; CRP, C-reactive protein; HR, hazard ratio; CI, confidence interval.

In both the CHARLS and ELSA cohorts, subgroup analyses were performed by age, sex, education, marital status, smoking, and drinking (Fig. 4).

In the CHARLS cohort, elevated MCMI showed a consistent positive association with CMM risk across all subgroups (all *P* < 0.05). Significant interactions were observed in the age, sex, education, smoking, and drinking subgroups (P for interaction < 0.05), indicating higher CMM risk among older individuals, males, those with higher education, smokers, and drinkers.

In the ELSA cohort, elevated MCMI remained positively associated with CMM risk across all subgroups except for education. Significant associations were observed in participants with high school education or above (HR = 1.90, 95% CI: 1.52–2.39), whereas no statistically significant association was found in participants with below high school education. No significant interactions were observed in the ELSA cohort.

### Predictive performance of MCMI for CMM

**Fig. 5:**
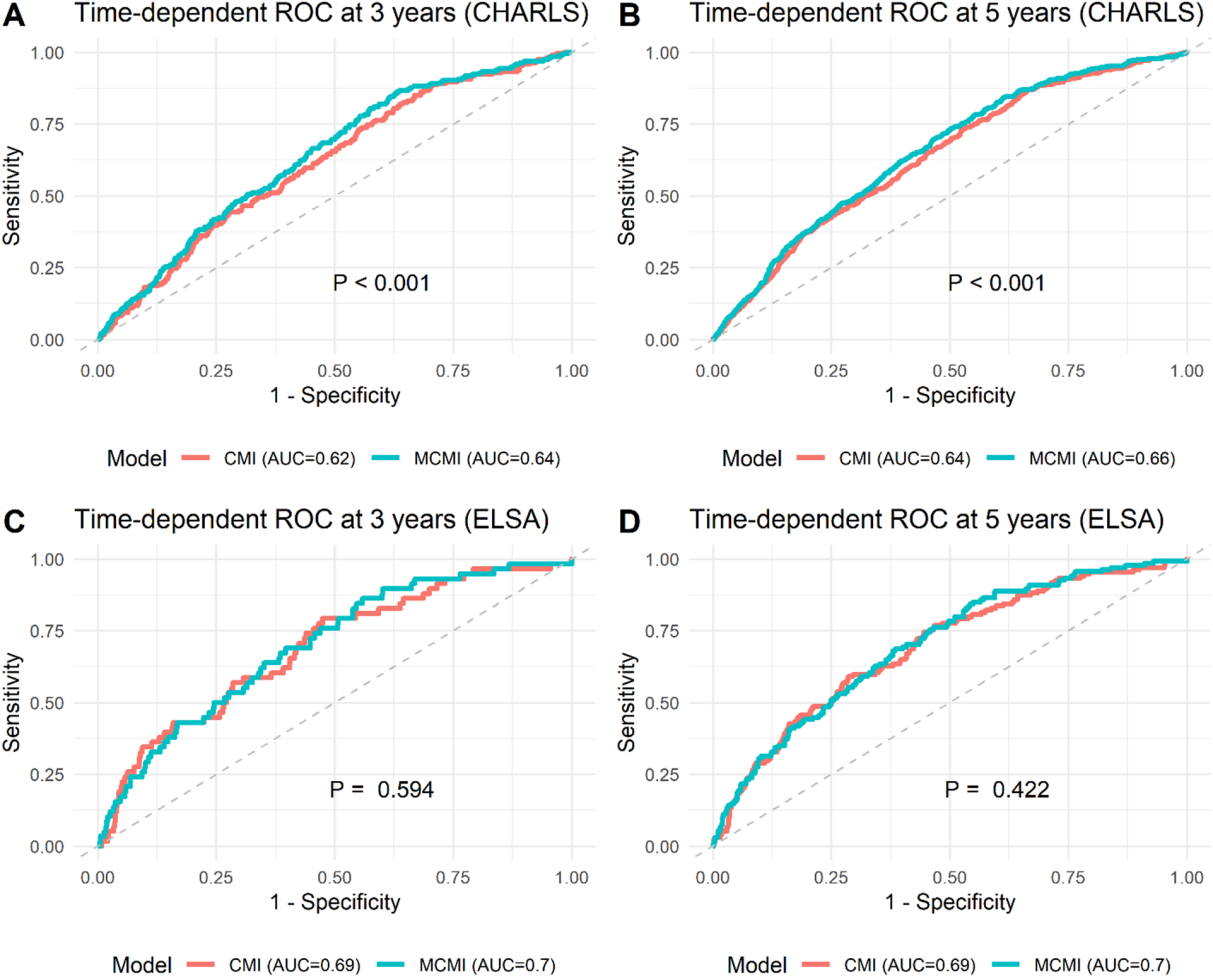
Time-dependent ROC curves for MCMI and CMI in CMM prediction. Panels A–B show the comparative predictive performance of MCMI and CMI for stroke at 3 and 5 years in the CHARLS cohort, while Panels C–D present the corresponding results in the ELSA cohort. All models were adjusted for age, sex, marital status, educational attainment, smoking, drinking, household income, LDL-C, CES-D score, and CRP. Abbreviations: MCMI, modified cardiometabolic index; CMI, cardiometabolic index; CHARLS, China Health and Retirement Longitudinal Study; ELSA, English Longitudinal Study of Ageing; CMM, cardiometabolic multimorbidity; LDL-C, low-density lipoprotein cholesterol; CES-D, Center for Epidemiologic Studies Depression Scale; CRP, C-reactive protein; ROC, receiver operating characteristic; AUC, area under the curve.

Time-dependent ROC analyses (Fig. 5) demonstrated that the MCMI model consistently achieved higher AUC values than the CMI model at both 3- and 5-year follow-up (3 years: CHARLS MCMI 0.64 vs. CMI 0.62; ELSA MCMI 0.70 vs. CMI 0.69; 5 years: CHARLS MCMI 0.66 vs. CMI 0.64; ELSA MCMI 0.70 vs. CMI 0.69), with detailed AUC estimates and 95% confidence intervals provided in Table S2. In the CHARLS cohort, DeLong tests indicated statistically significant differences at both time points (all *P* < 0.05), confirming the superior discriminative ability of MCMI. In contrast, the differences in the ELSA cohort were not statistically significant (all *P* > 0.05), indicating that MCMI did not demonstrate clear superiority over CMI in this population.

### Sensitivity Analyses

To evaluate the robustness of our findings, we performed several sensitivity analyses. First, owing to data limitations, physical activity was additionally adjusted only in the CHARLS cohort. Although this adjustment resulted in the exclusion of a considerable number of participants with missing data, the results remained consistent with the main analyses (Table S1, Figure S1). Second, proportional hazards assumptions were examined for Model 3 using Schoenfeld residuals, and all covariates showed non-significant test results (all *P* > 0.05), indicating no violation of the proportional hazards assumption (Table S3). Third, to address the potential confounding introduced by both the logarithmic transformation and the inclusion of FBG in the MCMI formula, we further constructed a log-transformed CMI (log-CMI = ln[TG/HDL-C] × WC/height) and compared its predictive performance with CMI and MCMI using time-dependent ROC analyses. In the CHARLS cohort, the AUC values at 3 years were 0.64 for MCMI, 0.63 for log-CMI, and 0.62 for CMI, and at 5 years were 0.66, 0.65, and 0.64, respectively (Figure S2, Figure S3). Pairwise comparisons showed that the differences between log-CMI and MCMI, as well as between log-CMI and CMI, were not statistically significant (all *P* > 0.05). These findings suggest that both the inclusion of FBG and the log-transformation contribute to improved performance, and the enhancement observed for MCMI likely reflects the combined influence of these two components. Lastly, we estimated E-values to examine the potential impact of unmeasured confounding. In the CHARLS cohort, the E-value was 1.67 for MCMI as a continuous variable and 7.08 for the highest versus lowest quartile comparison. In the ELSA cohort, the corresponding E-values were 2.87 and 4.98, respectively. These values suggest that substantial unmeasured confounding would be required to fully explain the observed associations, supporting the robustness of our results.

## Discussion

In this analysis of two large nationally representative cohorts (CHARLS and ELSA), higher MCMI was consistently associated with increased CMM risk. Dose–response patterns were evident across MCMI quartiles, with RCS analyses suggesting a nonlinear association in CHARLS and a linear trend in ELSA, highlighting potential population-specific differences. Subgroup analyses confirmed the robustness of associations across all subgroups in CHARLS, with significant interactions observed for age, sex, education, smoking, and drinking; in ELSA, associations were consistent across subgroups except for education. Importantly, MCMI demonstrated superior predictive performance over the conventional CMI model in the CHARLS cohort, whereas no significant difference was observed in ELSA. Sensitivity analyses, including additional lifestyle adjustment and E-value estimation, supported the robustness of these findings. Collectively, our study demonstrates that MCMI is an independent and incremental predictor of incident CMM across diverse populations.

CMI has been widely reported as a marker associated with multiple metabolic abnormalities and cardiovascular diseases. Prior studies have linked CMI to carotid atherosclerosis^28^, renal endothelial dysfunction^29^, hyperuricemia^30^, nonalcoholic fatty liver disease^31^, hypertension^32^, metabolic syndrome^33^, and insulin resistance^34^, as well as adverse outcomes including heart failure^35^, ischemic heart disease^13^, stroke^14^, and all-cause mortality^36^. Beyond cardiometabolic outcomes, CMI has also been examined in relation to obstructive sleep apnea^37^, depression^38^, and reproductive health disorders^39,40^.

With advancing age, multimorbidity becomes increasingly prevalent. An epidemiological study in Portuguese primary care centers reported that 72.7% of individuals had multiple chronic conditions^41^. Among these, CMM has received particular attention since its initial definition in 2015 due to its strong association with mortality risk^2^. More recently, CMM has emerged as a major public health challenge, especially during the COVID-19 pandemic, where it was strongly associated with poor prognosis^42^. Overweight and obesity are recognized as major risk factors for CMM^3^, while central adiposity measures, such as waist circumference and waist-to-height ratio, have been shown to outperform BMI in predicting CMM^43^. Consistently, CMI, which integrates waist-to-height ratio and lipid metabolism, has also been reported to be associated with CMM^15^.

Building on this evidence, MCMI was developed by incorporating FBG and applying a logarithmic transformation to the original CMI formula, aiming to better capture metabolic dysfunction. In the present study, we evaluated MCMI in both the CHARLS and ELSA cohorts and observed that higher MCMI levels were consistently associated with increased CMM risk, consistent with prior findings for CMI^15^. Time-dependent ROC analyses showed that MCMI provided better predictive performance than CMI across follow-up periods, with statistically significant improvements in the CHARLS cohort, although not in ELSA. Sensitivity analyses suggested that both the inclusion of FBG and the application of log-transformation may contribute to the improved predictive performance, with the observed enhancement for MCMI in CHARLS likely reflecting the combined influence of these two components. Taken together, these findings suggest that, particularly among Chinese middle-aged and older adults, MCMI may serve as a promising indicator for predicting CMM.

Subgroup analyses in the CHARLS and ELSA cohorts provided further insights into the association between elevated MCMI and CMM risk. In CHARLS, the positive association was consistent across all subgroups, with significant interactions observed for age, sex, education, smoking, and drinking, indicating higher CMM risk among older individuals, males, those with higher education, smokers, and drinkers. These findings are consistent with prior evidence suggesting that both biological and lifestyle factors can modify cardiometabolic risk^44,45^. In ELSA, associations were generally consistent and significant across all subgroups, except for education. Within the education subgroup, participants with high school education or above showed significant associations, whereas those with below high school education did not reach statistical significance, likely due to smaller sample size or reduced statistical power rather than a true absence of effect. Importantly, no significant interactions were observed in ELSA, suggesting that the effect of elevated MCMI on CMM risk is largely uniform across sociodemographic strata in this cohort. Collectively, these results indicate that cardiometabolic risk is influenced by both universal mechanisms and context-specific factors, emphasizing the need to consider population characteristics, including sociodemographic and cultural contexts, in risk assessment and prevention strategies.

From a mechanistic perspective, elevated MCMI reflects visceral fat accumulation and associated metabolic disturbances, which are closely linked to an increased risk of CMM. Dysfunctional visceral adipose tissue acts as an active inflammatory organ, continuously releasing free fatty acids and pro-inflammatory cytokines, thereby inducing systemic insulin resistance and chronic low-grade inflammation^46^. Insulin resistance promotes hypertension via renal sodium retention and sympathetic activation^47^, while lipotoxicity and inflammation impair pancreatic β-cell function, increasing the risk of diabetes^48^. Concurrently, insulin resistance, hypertension, chronic inflammation, and dyslipidemia synergistically impair endothelial function and accelerate atherosclerosis, providing a shared pathological basis for cardiovascular events and stroke^49^. Therefore, MCMI, by reflecting this pro-pathogenic milieu, may serve as a key indicator for predicting the synergistic development of multiple cardiometabolic diseases.

MCMI shows strong potential for clinical application. Its calculation relies on routinely collected, easily obtainable indicators—blood glucose, blood lipids, and waist-to-hip ratio—which are simple and cost-effective, making it well suited for population-level screening and primary care. We observed a positive, dose–response association between MCMI and CMM risk. Although RCS analysis revealed cohort-specific patterns—nonlinear in CHARLS and linear in ELSA—both support MCMI’s utility for risk stratification. In the CHARLS cohort, ROC analysis further indicated that MCMI outperformed traditional CMI in predicting CMM, suggesting improved discriminatory capacity. Subgroup analyses in CHARLS revealed significant interactions (*P* < 0.05), indicating that the association between higher MCMI levels and increased CMM risk was stronger among older individuals, men, those with higher educational attainment, smokers, and drinkers, underscoring its relevance for targeted risk evaluation. Nonetheless, as this study is based on a dual-cohort observational design, these findings require validation in independent cohorts.

This study has several notable strengths. By leveraging two large, nationally representative cohorts (CHARLS, *n* = 7,203; ELSA, *n* = 2,225), it ensured adequate sample size and statistical power. The dual-cohort design with cross-validation enhanced the robustness of the results, and the consistency of findings across distinct populations supports their generalizability. Nevertheless, some limitations should be acknowledged. First, the diagnosis of CMM relied on self-reported physician diagnoses, which may introduce recall and misclassification bias and potentially attenuate the observed associations. Nonetheless, prior validation studies have shown that self-reported cardiometabolic conditions demonstrate acceptable reliability^50,51^. Second, the exclusion of a substantial number of participants due to missing data may have led to selection bias and underestimated the true association between MCMI and CMM. Third, although multivariable adjustments were conducted in Model 3, residual confounding inherent to observational studies cannot be entirely ruled out. Fourth, while MCMI demonstrated strong predictive performance overall, statistical significance was not achieved in the ELSA cohort, which may limit its clinical applicability and warrants further investigation. Fifth, comparison between CMI and MCMI involves inherent methodological differences, as MCMI incorporates both fasting blood glucose and a logarithmic transformation, whereas CMI does not, and the indices use different measurement units (mmol/L vs. mg/dL). These factors may introduce analytical confounding and should be considered when interpreting comparative performance. Sixth, although this study provides valuable comparative evidence from Chinese and European populations, external validation in additional independent cohorts remains necessary to confirm the findings. Finally, although we analyzed two large, nationally representative cohorts, we did not pool the data due to substantial differences in population characteristics, laboratory measurements, outcome definitions, and follow-up durations. Direct pooling could introduce bias from cohort heterogeneity. Instead, parallel analyses were conducted in each cohort, allowing qualitative comparison of results. Future studies using standardized measurements across populations may permit pooled analyses to provide more generalized estimates.

## Conclusion

In conclusion, this study provides evidence that MCMI is positively associated with CMM in both the CHARLS and ELSA cohorts, with higher risk observed among older individuals, males, those with higher education, smokers, and drinkers in CHARLS. These findings highlight the need for tailored preventive strategies targeting these high-risk groups in China. RCS analyses revealed a nonlinear association in CHARLS but a linear trend in ELSA, suggesting potential population-specific differences that warrant further investigation. Moreover, in CHARLS, MCMI showed superior predictive performance for CMM compared with CMI, supporting its potential utility as a complementary indicator for CMM risk assessment in clinical and public health settings. Additional studies in diverse populations are needed to confirm these findings and elucidate underlying mechanisms.

## Supplementary Information

Below is the link to the electronic supplementary material.

Supplementary Material 1

Supplementary Material 2

Supplementary Material 3

Supplementary Material 4

Supplementary Material 5

Supplementary Material 6

Supplementary Material 7

## Data Availability

The data analyzed in this study are openly accessible from the CHARLS repository (http://charls.pku.edu.cn/en) and the UK Data Service (https://beta.ukdataservice.ac.uk/). Analytical outputs generated during the study are available from the corresponding author on reasonable request.
