# Supplementary material for: Association between modified cardiometabolic index and cardiometabolic multimorbidity in middle-aged and older adults: evidence from two nationwide cohort studies
Source: Sci Rep. 2026 Feb 23;16:10274. doi: 10.1038/s41598-026-41398-2 (PMC13031912; doi:10.1038/s41598-026-41398-2)
Supplement: Supplementary file 1 — Supplementary Material 1 [file 41598_2026_41398_MOESM1_ESM.docx]

| **Variable** | **Model1 HR(95% CI)** | **P value** | **Model2 HR(95% CI)** | **P value** | **Model3 HR(95% CI)** | **P value** |
| --- | --- | --- | --- | --- | --- | --- |
| MCMI (pre 1-unit) | 1.37 (1.30, 1.44) | < 0.001 | 1.38 (1.30, 1.45) | < 0.001 | 1.36 (1.26, 1.47) | < 0.001 |
| Quartiles of MCMI |  |  |  |  |  |  |
| Q1 (MCMI < 2.44) | Ref |  | Ref |  | Ref |  |
| Q2 (2.44 ≤ MCMI < 2.86) | 1.59 (1.17, 2.16) | 0.003 | 1.60 (1.18, 2.18) | 0.003 | 1.46 (1.07, 1.99) | 0.018 |
| Q3 (2.86 ≤ MCMI < 3.33) | 2.32 (1.74, 3.10) | < 0.001 | 2.39 (1.78, 3.19) | < 0.001 | 1.96 (1.46, 2.64) | < 0.001 |
| Q4 (MCMI ≥ 3.33) | 4.42 (3.37, 5.79) | < 0.001 | 4.52 (3.43, 5.94) | < 0.001 | 2.96 (2.22, 3.96) | < 0.001 |
| P for trend |  | < 0.001 |  | < 0.001 |  | < 0.001 |
