# Supplementary material for: Association between modified cardiometabolic index and cardiometabolic multimorbidity in middle-aged and older adults: evidence from two nationwide cohort studies
Source: Sci Rep. 2026 Feb 23;16:10274. doi: 10.1038/s41598-026-41398-2 (PMC13031912; doi:10.1038/s41598-026-41398-2)
Supplement: Supplementary file 2 — Supplementary Material 2 [file 41598_2026_41398_MOESM2_ESM.docx]

| Variable | Time-dependent ROC at 3 years (CHARLS)  AUC (95% CI) | Time-dependent ROC at 5 years (CHARLS)  AUC (95% CI) | Time-dependent ROC at 3 years (ELSA)  AUC (95% CI) | Time-dependent ROC at 5 years (ELSA)  AUC (95% CI) |
| --- | --- | --- | --- | --- |
| MCMI | 0.64 ( 0.60 , 0.68 ) | 0.66 ( 0.63 , 0.68 ) | 0.70 ( 0.63 , 0.76 ) | 0.70 ( 0.66 , 0.75 ) |
| CMI | 0.62 ( 0.58 , 0.65 ) | 0.64 ( 0.62 , 0.66 ) | 0.69 ( 0.62 , 0.76 ) | 0.69 ( 0.65 , 0.74 ) |
