# Supplementary material for: Association between modified cardiometabolic index and cardiometabolic multimorbidity in middle-aged and older adults: evidence from two nationwide cohort studies
Source: Sci Rep. 2026 Feb 23;16:10274. doi: 10.1038/s41598-026-41398-2 (PMC13031912; doi:10.1038/s41598-026-41398-2)
Supplement: Supplementary file 3 — Supplementary Material 3 [file 41598_2026_41398_MOESM3_ESM.docx]

| Variable (schoenfeld residual test) | Chisq (CHARLS) | P value (CHARLS) | Chisq (ELSA) | P value (ELSA) |
| --- | --- | --- | --- | --- |
| MCMI | 0.726 | 0.394 | 0.005 | 0.946 |
| Age | 0.072 | 0.789 | 3.484 | 0.062 |
| Gender | 0.013 | 0.909 | 0.025 | 0.874 |
| Marital status | 0.016 | 0.899 | 0.231 | 0.631 |
| Education | 0.952 | 0.329 | 0.753 | 0.386 |
| Smoking | 0.152 | 0.697 | 0.005 | 0.944 |
| Drinking | 0.038 | 0.845 | 1.391 | 0.238 |
| LDL-C | 0.015 | 0.904 | 3.710 | 0.054 |
| CES-D score | 0.527 | 0.468 | 0.256 | 0.613 |
| CRP | 0.552 | 0.458 | 2.461 | 0.117 |
| Household income | 0.197 | 0.657 | 0.134 | 0.715 |
| Global | 3.963 | 0.971 | 13.507 | 0.262 |
