# Supplementary figures and images for: Association between modified cardiometabolic index and cardiometabolic multimorbidity in middle-aged and older adults: evidence from two nationwide cohort studies
Source: Sci Rep. 2026 Feb 23;16:10274. doi: 10.1038/s41598-026-41398-2 (PMC13031912; doi:10.1038/s41598-026-41398-2)

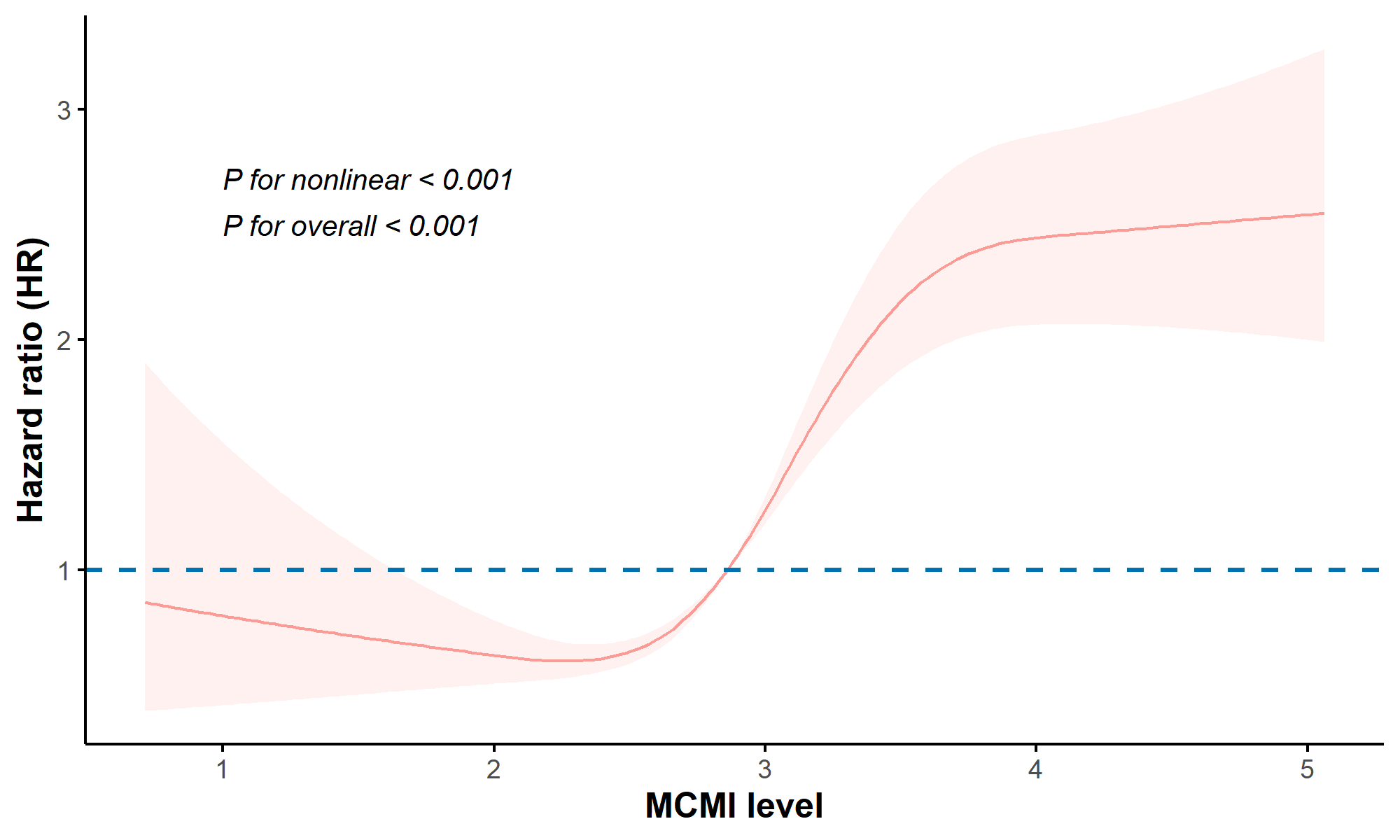

Supplement: Supplementary file 4 — Supplementary Material 4 [file 41598_2026_41398_MOESM4_ESM.tiff]

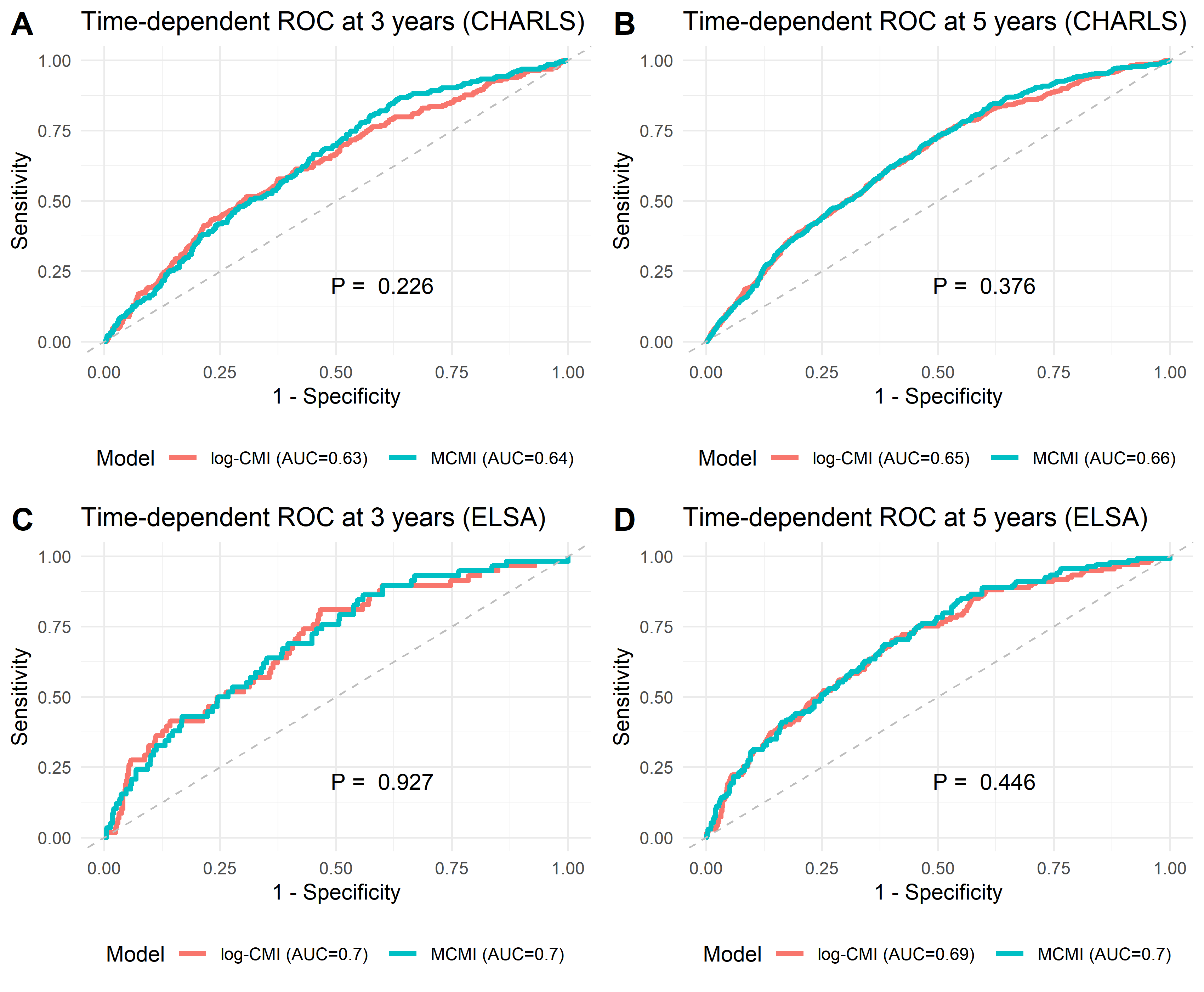

Supplement: Supplementary file 5 — Supplementary Material 5 [file 41598_2026_41398_MOESM5_ESM.tiff]

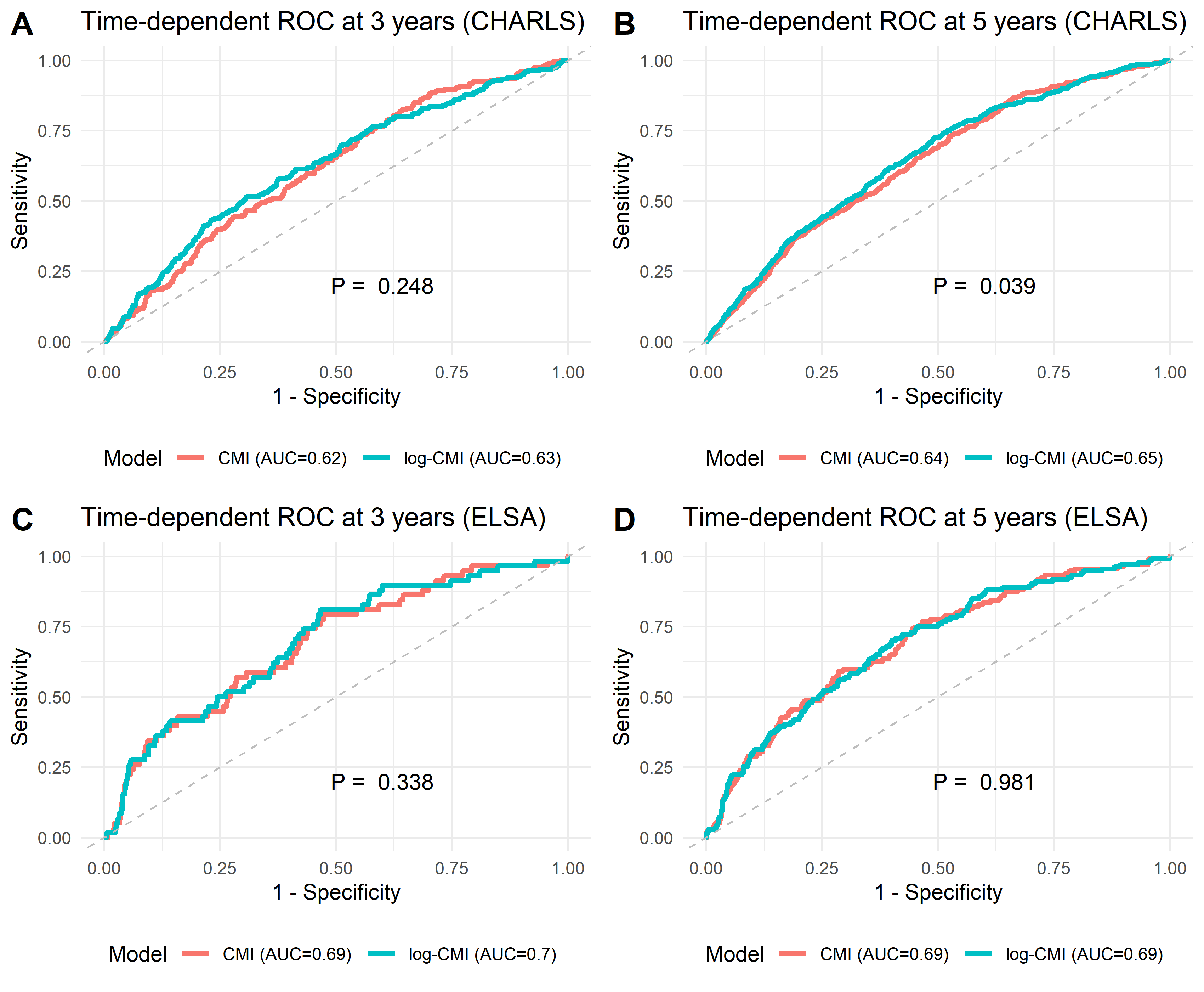

Supplement: Supplementary file 6 — Supplementary Material 6 [file 41598_2026_41398_MOESM6_ESM.tiff]
