# Supplementary material for: Association between modified cardiometabolic index and cardiometabolic multimorbidity in middle-aged and older adults: evidence from two nationwide cohort studies
Source: Sci Rep. 2026 Feb 23;16:10274. doi: 10.1038/s41598-026-41398-2 (PMC13031912; doi:10.1038/s41598-026-41398-2)
Supplement: Supplementary file 7 — Supplementary Material 7 [file 41598_2026_41398_MOESM7_ESM.docx]

Figure S1. Dose–response relationship between MCMI and CMM risk in the CHARLS cohort, additionally adjusted for physical activity

Restricted cubic spline showing adjusted hazard ratios for CMM across MCMI levels. The model was adjusted for age, sex, marital status, educational attainment, smoking, drinking, household income, LDL-C, CES-D score, CRP and physical activity.

Abbreviations: CHARLS, China Health and Retirement Longitudinal Study; CMM, cardiometabolic multimorbidity; MCMI, modified cardiometabolic index; LDL-C, low-density lipoprotein cholesterol; CES-D, Center for Epidemiologic Studies Depression Scale; CRP, C-reactive protein

Figure S2. Time-dependent ROC curves for MCMI and log-CMI in CMM prediction

Panels A–B show the comparative predictive performance of MCMI and log-CMI for stroke at 3 and 5 years in the CHARLS cohort, while Panels C–D present the corresponding results in the ELSA cohort. All models were adjusted for age, sex, marital status, educational attainment, smoking, drinking, household income, LDL-C, CES-D score, and CRP.

Abbreviations: MCMI, modified cardiometabolic index; CMI, cardiometabolic index; CHARLS, China Health and Retirement Longitudinal Study; ELSA, English Longitudinal Study of Ageing; CMM, cardiometabolic multimorbidity; LDL-C, low-density lipoprotein cholesterol; CES-D, Center for Epidemiologic Studies Depression Scale; CRP, C-reactive protein; ROC, receiver operating characteristic; AUC, area under the curve

Figure S3. Time-dependent ROC curves for CMI and log-CMI in CMM prediction

Panels A–B show the comparative predictive performance of CMI and log-CMI for stroke at 3 and 5 years in the CHARLS cohort, while Panels C–D present the corresponding results in the ELSA cohort. All models were adjusted for age, sex, marital status, educational attainment, smoking, drinking, household income, LDL-C, CES-D score, and CRP.

Abbreviations: CMI, cardiometabolic index; CHARLS, China Health and Retirement Longitudinal Study; ELSA, English Longitudinal Study of Ageing; CMM, cardiometabolic multimorbidity; LDL-C, low-density lipoprotein cholesterol; CES-D, Center for Epidemiologic Studies Depression Scale; CRP, C-reactive protein; ROC, receiver operating characteristic; AUC, area under the curve

Table S1. Sensitivity analyses of the association between MCMI and CMM risk in the CHARLS cohort, additionally adjusted for physical activity

Model 1: Unadjusted. Model 2: Adjusted for age, sex, marital status, educational attainment, smoking, drinking. Model 3: Additionally adjusted for household income, LDL-C, CES-D score, CRP and physical activity.

Abbreviations: CHARLS, China Health and Retirement Longitudinal Study; CMM, cardiometabolic multimorbidity; LDL-C, low-density lipoprotein cholesterol; CES-D, Center for Epidemiologic Studies Depression Scale; CRP, C-reactive protein; MCMI, modified cardiometabolic index; HR, hazard ratio; CI, confidence interval

Table S2. AUC values and 95% confidence intervals for MCMI and CMI in CMM prediction at 3 and 5 years

Abbreviations: CHARLS, China Health and Retirement Longitudinal Study; ELSA, English Longitudinal Study of Ageing; CMM, cardiometabolic multimorbidity; MCMI, modified cardiometabolic index; CI, confidence interval; CMI, cardiometabolic index; AUC, area under the curve

Table S3. Schoenfeld residual tests for proportional hazards assumption in Model 3 for the association between MCMI and CMM in the CHARLS and ELSA cohorts

Abbreviations: CHARLS, China Health and Retirement Longitudinal Study; ELSA, English Longitudinal Study of Ageing; CMM, cardiometabolic multimorbidity; MCMI, modified cardiometabolic index; LDL-C, low-density lipoprotein cholesterol; CES-D, Center for Epidemiologic Studies Depression Scale; CRP, C-reactive protein
